# Workshop report: Can an understanding of the mechanisms underlying age-related loss of muscle mass and function guide exercise and other intervention strategies?

**DOI:** 10.1186/2046-2395-1-5

**Published:** 2012-10-01

**Authors:** Malcolm J Jackson, Anne McArdle, Aphrodite Vasilaki, Anna Kayani

**Affiliations:** 1 Institute of Ageing and Chronic Disease, University of Liverpool, Liverpool, L69 3GA, UK

## Abstract

An international workshop was hosted by the University of Liverpool on 15–16 July 2011 to address at a basic level what is known about the fundamental mechanisms by which skeletal muscle mass and function are lost during aging and to examine the nature of interventions that might prevent these mechanistic changes. Of particular importance was to attempt to evaluate how different forms of exercise (or muscle contractile activity) influence these processes and how these effects can be best optimized to prevent or delay age-related loss of muscle function. The program took the form of a two-day meeting, comprising a series of invited talks and breakout sessions designed to identify key gaps in current knowledge and potential future research questions. The aims of this Workshop were two-fold: 1. To identify the current state-of-the-art in the understanding of the mechanisms that contribute to loss of skeletal muscle mass and function that occurs with aging and to address at a mechanistic level how, and to what extent, exercise and/or other interventions might prevent these changes. 2. To identify specific areas of research where information is sparse but which are likely to yield data that will impact on future strategies to manipulate age-related loss of muscle mass and function in older people. The areas discussed in detail were loss of functional motor units, reduced muscle stem cell activity, age-related changes in transcriptional responses of muscle to exercise and nutrition, age-related changes in protein homeostasis, mitochondrial function, altered cross-talk between muscle with immune cells and how the developments in basic science to understand mechanisms underlying age-related loss of muscle mass and function can be translated. Following each session three key areas where further studies are needed were identified.

## Meeting report

An international workshop was hosted by the University of Liverpool on 15–16 July 2011 to address at a basic level what is known about the fundamental mechanisms by which skeletal muscle mass and function are lost during aging and to examine the nature of interventions that might prevent these mechanistic changes. Of particular importance was the attempt to evaluate how different forms of exercise (or muscle contractile activity) influence these processes and how these effects can be best optimized to prevent or delay age-related loss of muscle function.

The program took the form of a two-day meeting, comprising a series of invited talks and breakout sessions designed to identify key gaps in current knowledge and potential future research questions.

The aims of this Workshop were two-fold:

1. To identify the current state-of-the-art in the understanding of the mechanisms that contribute to loss of skeletal muscle mass and function that occurs with aging and to address, at a mechanistic level, how and to what extent, exercise and/or other interventions might prevent these changes.

2. To identify specific areas of research where information is sparse but which are likely to yield data that will impact on future strategies to manipulate age-related loss of muscle mass and function in older people.

The workshop received financial support from the Biotechnology and Biological Sciences Research Council (BBSRC), Age UK and the University of Liverpool. Sixty participants attended together with 15 invited speakers.

## Background to the topic

Age-related loss of skeletal muscle mass and function is a major cause of loss of mobility, increased frailty and falls in older people and impacts profoundly on the quality of life of older people. By 70 years of age, muscle cross-sectional area (CSA) is reduced by 25 to 30% due to a loss of muscle fibers and atrophy of the remaining fibers. These deficits profoundly impact on the quality of life of even healthy older people, as many are at or near functionally relevant strength thresholds that limit the ability to carry out everyday tasks. Age-related muscle weakness increases the potential for falls and many older people who fall suffer loss of independence and some never re-enter the community. The loss of muscle fibers is the major component responsible for the reduced force generation by muscle which occurs in older humans [1-21].

Most approaches to improving muscle function in aged humans or animals have tried to improve the function of the residual muscle fibers present in older people using exercise training regimens or nutritional enhancements. Clearly some exercise regimens can improve muscle function and mass in older subjects although the effects are diminished in older people compared with younger subjects. Thus, although exercise regimes improve muscle function at all ages by hypertrophy of existing/remaining muscle fibers, they do not appear to influence the loss of fibers and hence even active older adults, including veteran athletes, still develop an age-related decline in muscle mass and function (for example, [22-24]). The current situation is therefore that interventions may seek to optimize the mass and function of residual muscle in older people, but do not specifically address the underlying loss of muscle fibers or attempt to replace lost fibers.

The precise mechanisms by which muscle fibers are lost with advancing age are relatively poorly understood, although there is increasing evidence that a number of mechanisms may play a role. For this workshop these were grouped into six topic areas with two invited presentations for each of the areas with speakers being invited to provide their personal view of the state-of-the-art in each of these areas. Following the presentations, open discussion sessions were held to consider each of the areas in more detail and to attempt to agree to at least three key points where information is sparse but which are likely to yield data that will impact on future strategies to manipulate age-related loss of muscle mass and function in older people. The topics were:

## Loss of functional motor units

Clear data indicate that loss of motor neurons accompanies the loss of muscle fibers [25-30] but whether this process drives the loss of fibers or is secondary to it is not known, as is whether any practical means of prevention of this loss have been identified. Dr. Lars Larsson (Uppsala University, Sweden) and Dr. Susan Brooks (University of Michigan) presented evidence that motor units are lost during aging and addressed whether physical exercise can prevent loss of motor units. Following the break out session, three key areas where further studies are needed were identified as:

Defining whether motor neuron loss during aging is intrinsic to the neuron.

Defining the importance of the compensatory sprouting that occurs and whether the process can be influenced by interventions.

Defining the factors that influence peak motor neuron numbers in development and how this influences motor neuron loss with aging.

## Reduced muscle stem cell activity

Data on the role of muscle stem cells (satellite cells) in age-related changes in muscle indicate that restoration of stem cell proliferative capacity may restore the regenerative potential in muscle from old animals and prevent age-related loss of fibers [31], but the overall relevance of this process to the loss of muscle fibers is controversial and approaches to translation of this work are not immediately apparent. Dr. Vincent Mouly (Institute de Myologie, Paris) and Dr. Stephen Harridge (Kings College, London) gave talks on the mechanisms of loss of stem cell function in muscle with aging and whether exercise or physical activity can influence stem cell activity in muscle. Data suggest that modified muscle stem cell function and behavior occurs with age, although the impact of this on muscle function and the effect of modified environment are controversial [32]. Following the break out session, three key areas where further studies are needed were identified as:

Understanding the availability and functionality of stem cells in old muscle and defining the effect of stress and responses to stress in muscle stem cells.

Determining the function of muscle stem cells under chronic stress. What can we learn from disease models?

Examining the behavior of stem cells *in vitro* compared with *in vivo* (interactions with environment, cross talk with interstitium, immune cells).

## Age-related changes in transcriptional responses of muscle to exercise and nutrition

Many studies show that the ability of tissues from older people to respond to stimuli is attenuated and data indicate that this includes an inability of muscle to respond to contractile activity or nutrition. Some transgenic studies have shown that this inability to respond may play a fundamental role in the loss of muscle mass and function, but the overall relevance of this area to loss of muscle mass and function in humans is unclear. Dr. Andrea Musterberg (University of East Anglia) and Dr. Anne McArdle (University of Liverpool) gave talks on microRNAs in skeletal muscle development and differentiation and the role of physical activity in overcoming lack of responsiveness. Data suggest that further work is necessary to characterize the role of miRNAs. In addition, the mechanisms by which muscles of older individuals are relatively resistant to adaptive responses are unclear [33]. Following the break out session, three key areas where further studies are needed were identified as:

What is the nature, time course, role and targets of miRNAs in aging muscle and how are they influenced by environmental factors such as exercise or the immune system (is the response of the transcriptome longitudinally stable in muscle – are the effects of circadian rhythm, season, eating of the same magnitude?).

What are the mechanisms responsible for a failure of adaptation in muscles of older individuals and what are the differences between responders and non-responders, for example, gender differences, the role of immune system?

To what extent can such changes be attributed to disuse?

## Age-related changes in protein homeostasis

Dr. Marco Sandri (University of Padova, Italy) and Dr. Anton Wagenmakers (University of Birmingham) gave talks on the changes in muscle protein homeostasis with aging and the role of activity and anabolic agents in regulating protein turnover in aging. Data suggest that changes in muscle protein synthesis and/or breakdown have been widely reported to accompany the muscle atrophy that occurs in aging (for example, [34]) but the precise nature of these changes is the subject of considerable controversy that accompanies an important debate about the relevance of rodent models in this area [35]. Resistance training to induce hypertrophy and nutritional supplements are widely advocated as a means of increasing muscle protein accretion inolder people. Following the break out session, three key areas where further studies are needed were identified as:

How do we identify synthesis or degradation as the initiating factor in loss of muscle mass and function – for example, what is the impact of intervention on motor unit loss; what is the role of inflammation on protein homeostasis?

Need to understand model and technique correlations and differences. For example, using mice as a model system for humans, what are the questions that can and cannot be answered in human studies with the available techniques? Can novel techniques be developed in humans?

To what extent can changes in protein homeostasis be attributed to disuse?

## Altered mitochondrial function

Dr. David Hood (York University, Canada) and Dr. Doug Turnbull (Newcastle University) summarized altered mitochondrial function, content and biogenesis in aging muscle and whether mitochondrial changes during aging can be influenced by exercise. Many studies indicate that abnormalities in mitochondrial function occur in tissues with aging (for example, [36-38]) and several putative interventions are aimed at correction of these processes. Physiological data argue against a major role for mitochondrial dysfunction in the loss of muscle function, but basic transgenic interventions tend to support a key role for mitochondria. Exercise interventions to stimulate mitochondrial biogenesis are widely proposed as a practical means of overcoming these putative defects in muscle, but the relevance of these are unclear. Following the break out session, three key areas where further studies are needed were identified as:

Defining whether the mitochondrial defects are primary events during aging and whether they are inevitable

Defining whether mitochondria deficits are important in motor neuron loss during aging

Defining whether muscle cytochrome oxidase deficiency seen in some fibers on biopsy leads to loss of the fibers.

## Altered cross-talk between muscle and immune cells

Dr. Michael Reid (University of Kentucky, USA) and Dr. Hans Degens (Manchester Metropolitan University) summarized the role of systemic inflammation in age-related muscle weakness and wasting. Aging is associated with a chronic pro-inflammatory state and data indicate that inflammation may influence muscle force generation, regeneration from damage and other aspects of the aged muscle phenotype [39,40]. Recent data indicate a crucial role for muscle and exercise in regulating the pattern of circulating inflammatory mediators and that substantial cross-talk occurs between muscle and the immune system, although how this is modified during aging is unclear.

Following the break out session, three key areas where further studies are needed were identified as:

Determining what defines the pro-inflammatory state reported in older people and whether this is influenced by exercise

Determining how much of the aging-related inflammation is intrinsic to the muscle or due to soluble mediators

Determining whether the muscle inflammation is related to cross-talk with non-immune cells, such as adipocytes.

The final session at the workshop looked at how to translate the developments in basic science to understand mechanisms underlying age-related loss of muscle mass and function. Dr. Avan Sayer (University of Southampton) and Dr. Carolyn Greig (University of Edinburgh) gave talks on factors causing loss of muscle mass in people compared with experimental models and approaches to prevention and practical interventions to maintain muscle mass in older people. In discussion, the following aspects were felt to be important in developing translational research:

What are the effects of long term feeding studies in older people, since too many acute feeding studies have been undertaken for which the relevance is inconclusive?

Are there practical strategies to translate basic research on how muscle fibers are lost during aging into practical recommendations for exercise inolder people?

More communication is needed between researchers using animal and human models to facilitate translation and resolve controversies regarding relevance of different experimental models.

Understanding the psychology of behavioral change is a priority. There is a need for more information on how to influence motivation, why older people do less exercise and what affects compliance with exercise regimens? What social, cultural and psychological factors influence lifetime exercise and how can advice be improved considering these issues? Is what is needed simple, effective dietary and exercise advice? Issues, such as making exercise fun and accessible and ensuring building infrastructure is appropriate to encourage exercise, are important. The group queried how people could be encouraged to take personal responsibility for maintaining health through exercise and so on,whether guidelines are effective.

The UK has a number of important established population cohorts and it is important to maximize the benefit of these for research in this area. There was also a recognition that more could be learned from studies undertaken in groups of humans who were aging “well”, such as centenarians or masters athletes.

Muscle loss and weakness is a major societal challenge and the impetus to encourage exercise may benefit from a cost-benefit analysis examining whether money spent on encouraging exercise can lead to savings in future health budgets.

At a basic science level there is a need to understand what defines individual responses to exercise and, in this, studies with out-bred animal models may aid understanding, as may human genetic studies.

We would like to thank BBSRC and AgeUK for their generous financial support of this workshop.

## Competing interests

The authors declare that they have no competing interests.

## Authors’ contribution

MJJ and AM identified the need for a focused Workshop, MJJ, AM, AV and AK established the program and hosted the Workshop. All authors read and approved the final manuscript.
